# Commentary: Comparison of radiological interpretation made by veterinary radiologists and state-of-the-art commercial AI software for canine and feline radiographic studies

**DOI:** 10.3389/fvets.2025.1615947

**Published:** 2025-06-25

**Authors:** Steve K. Joslyn, Josephine Faulkner, Doris Ma, Ryan Appleby

**Affiliations:** ^1^School of Veterinary and Life Sciences, Murdoch University, Perth, WA, Australia; ^2^Department of Clinical Studies, Ontario Veterinary College, University of Guelph, Guelph, ON, Canada

**Keywords:** artificial intelligence-AI, radiology, veterinary, study design and best practice, critical review

## Introduction

Ndiaye et al. report a head-to-head comparison of a commercial artificial intelligence radiology software (AI) and veterinary radiologists interpreting canine and feline radiographs (1). The conclusion states that the AI “performs almost as well as the best veterinary radiologist in all settings of descriptive radiographic findings” (1). The AI showed high specificity (ability to correctly identify normal findings) but lower sensitivity (ability to correctly detect abnormal findings) than radiologists (1), leading the authors to suggest that its strength lies in confirming normal cases. It is further postulated that the AI's performance is comparable to human experts and that the AI “will likely complement rather than replace human experts” in veterinary radiology (1). This is a noteworthy finding given the scarcity of veterinary radiologists, and the prospect of the AI aiding or augmenting clinical practice. However, a closer examination reveals several methodological and interpretive limitations that temper these optimistic conclusions. This commentary discusses the most impactful concerns—from the study's lack of a true gold-standard reference and biased sample, to statistical and ethical issues—and highlights their implications for real-world veterinary radiology.

## Methodological limitations in study design

### Absence of an independent ground truth

The study did not validate the AI interpretations against any definitive gold standard such as surgical/pathology findings or long-term clinical outcomes. Instead, “ground truth” was effectively defined by consensus of the observers themselves (1). Specifically, each radiographic finding was determined to be “normal” or “abnormal” based on the majority (median) opinion of the participating radiologists (mean of <6 per case), but also that of the AI (1, 2). The ground truth should be independent from the variable being evaluated, so including the AI's own output in establishing the correct answer is a form of circular logic—the tool being evaluated helps decide whether its prediction is considered correct. Even aside from that, radiologist consensus is an inherently imperfect reference standard (3). In this study, there was no independent verification of which interpretations were actually correct in a clinical sense, nor were radiologist interpretations independently and transparently coded, casting further doubt on the reliability of the reference standard. This makes it impossible to know whether the AI or human readers were “right for the right reasons” since discrepancies were never resolved by a definitive test. The lack of an external ground truth substantially limits confidence in claims of AI accuracy or radiologist error rates.

### Small, potentially biased sample with class imbalance

The experiment analyzed just 50 radiographic cases (40 canine, 10 feline), retrospectively selected from a single institution's PACS with no power calculation or other reasoning given for the number, and the paper acknowledges the limitation of this relatively small sample that may not capture the full spectrum of cases in veterinary practice (1). Moreover, the composition of findings was severely skewed: 84% of all reported findings were determined on consensus to be normal and only 16% abnormal (1). This imbalance (more than five normal for every abnormal) can inflate performance metrics and mask weaknesses. For instance, overall accuracy is high in part because a large majority of cases had no lesions—the authors even note that a naive strategy of calling everything “normal” would be correct 84% of the time on this dataset (1). Indeed, one of the participating radiologist's accuracy was not significantly better than this trivial baseline (1). Furthermore, the fact that each radiologist did not interpret every case introduces potential bias—particularly if lower-performing radiologists reviewed more cases—further undermining the validity of performance comparisons. In such a scenario, AI can achieve impressive accuracy and specificity simply by exploiting the class imbalance (leaning toward frequent “normal” outputs), without truly demonstrating robust abnormality detection. High sensitivity is essential for ruling out disease and is a critical requirement for any screening test (4, 5). However, the AI demonstrated overall low sensitivity (0.688), which declined further in both low-ambiguity (0.578) and high-ambiguity (0.444) settings (1). Therefore, the authors' conclusion suggesting the use of AI as a screening tool is contradictory. Additionally, the low prevalence of abnormal cases here means the study provides limited insight into the AI's ability to detect the diverse pathologies a veterinarian might encounter (6, 7). Lastly, the small case numbers weaken the evidence for broad clinical equivalence between the AI and radiologists (6).

### Ambiguity and *post-hoc* stratification

The study methodology introduces the concept of “low-ambiguity” vs. “high-ambiguity” cases based on the overall agreement among all observers. Variance in assessments was computed between each radiologist and the AI for every finding and used to categorize findings as ambiguous or not (1). While exploring performance on easy vs. difficult cases is a valid aim, the manner in which ambiguity was defined raises concerns. Firstly, the study includes a methodological choice to classify “insignificant abnormalities” as abnormal, a decision that introduces potential misalignment with clinical practice, where incidental or clinically irrelevant findings are often disregarded (5, 8). Importantly, this assumption was applied only to radiologists' interpretations; the AI output was consistently explicit in labeling findings as normal or abnormal. Thus, radiologists who naturally ignored these insignificant findings—as they commonly do in clinical practice—would appear to have artificially “missed” abnormalities. This likely resulted in an artificial reduction in diagnostic utility for radiologists compared to the AI, negatively affecting their relative statistical outcomes and perceived diagnostic performance (5). Secondly, by deriving ambiguity from the overall inter-observer agreement—which includes the AI's input—the metric may conflate case difficulty with observer performance, thus casting doubt on its validity as a true measure of ambiguity. Additionally, the original study failed to explicitly define what constitutes a “finding,” provide a comprehensive list of possible findings, or clearly specify how many findings the AI detected, further complicating this measure. In other words, a low consensus may reflect either truly ambiguous radiographs or cases where the AI (and potentially some radiologists) misinterpreted straightforward findings. Using this approach, the authors report that the AI “did better than the median radiologist overall in low- and high-ambiguity cases” (1) but this could be an artifact of the classification method. Truly evaluating AI on “challenging” cases would require an independent measure of case difficulty or ambiguity (for example, cases with known subtle lesions, or confirmed diagnoses that radiologists often miss), rather than one derived solely from the observers' agreement. Therefore, conclusions about the AI's reliability in high-ambiguity scenarios should be viewed with skepticism—they may not generalize beyond this specific sample and methodology.

### Inadequate handling of correlated data and statistics

The study design involved multiple readings of the same cases–11 radiologists interpreted many of the same 50 cases, generating over 16,000 individual “findings” data points (1). This is often termed a multi-reader, multi-case (MRMC) study, where the data is cross-correlated—results from the same case and the way in which one observer reads all cases are likely to be related (9). The authors employed a z-test for proportions to evaluate differences in diagnostic performance across observers. However, this is a basic statistical test which does not account for clustering of data: the readings are clustered by case (the same abnormality or normal finding was assessed by multiple readers) and by reader (each radiologist contributed many data points). Additionally, the z-test assumes that all observations are independent. Instead, each “normal” or “abnormal” finding represented repeated observations (1), violating a core assumption of the z-test. By using the z-test, the statistical significance reported is likely overstated.

In MRMC study designs, more sophisticated statistical methods are warranted, such as generalized estimating equations (GEEs), two-family gatekeeping approach, and bootstrapping (4, 9). Another novel method comprises a three-part regression model, based on the lesion detection rate, normal subject misdiagnosis rate (i.e., false positive), and the area under the conditional free response operator curve (measures how well the reader can differentiate between a true and false positive for a lesion) (9). In practical terms, the lack of statistical rigor means we should be cautious in accepting statements such as “the AI was no less accurate than any radiologist” as proven fact—especially given the small number of unique cases (6). A subtle difference in performance could easily be missed or poorly estimated. For example, the study found no statistically significant difference between the AI and the single best radiologist (*p* ≈ 0.08 in one metric), and declared them equivalent, but with only 50 cases this could simply reflect limited power rather than true equality (1, 10). Robust conclusions about non-inferiority would require a larger sample and appropriate analysis (11).

Beyond the core methodological limitations, the manuscript makes several interpretive claims that warrant further scrutiny. For instance, the assertion that “AI is likely to exhibit less random variation than an equally accurate human expert” is presented as a general truth, yet no empirical evidence is provided to support this statement in the context of the specific AI system evaluated. This raises concerns about overgeneralization. These choices, combined with a lack of external validation, underscore the need for caution when interpreting claims of AI consistency or superiority, particularly in real-world veterinary settings where clinical relevance and diagnostic precision are critical (6).

## Ethical and clinical implications of overstating AI performance

Even with the limitations, the authors of the study are optimistic about the AI's clinical utility, suggesting that “broader use of AI could reliably increase diagnostic availability” in veterinary medicine, provided humans remain in the loop (1). The idea of augmenting scarce radiology services with AI is compelling, however, it is ethically imperative to avoid overstating the readiness and capabilities of AI based on limited evidence. Given the fundamental flaws highlighted—lack of independent ground truth, small sample size, methodological biases, and inadequate statistical analysis—the study's conclusions are highly questionable, necessitating extreme caution regarding clinical uptake. Over-enthusiastic interpretation of results could mislead practitioners or policymakers into adopting AI tools prematurely or with insufficient oversight. As of March 2024, SignalRAY^®^ services more than 2,300 clinics world-wide (12), and there are anecdotal reports of this reaching 3,000 clinics already in 2025. As uptake of AI services continues to grow, premature acceptance based on methodologically flawed studies poses significant ethical and clinical risks.

Publications claiming AI to be “as good as or better than experts” are becoming increasingly common, often amplified by media hype, despite notable methodological flaws (13, 14). A 2020 British Medical Journal systematic review reported that over 70% of such publications found AI performance on par with clinicians, yet most were at high risk of bias and did not adhere to reporting standards (14). The review cautioned that “arguably exaggerated claims” about AI's equivalence to human experts pose risks to patient safety and population health, further warning that overpromising language can mislead the media and public into accepting inappropriate care (14). These concerns directly apply here: if one takes Ndiaye et al.'s findings at face value—that an off-the-shelf AI can essentially match a radiologist—then clinics, particularly those lacking specialist support, might rely too heavily on AI for primary interpretations. Yet, as discussed, such reliance could lead to missed diagnoses or case mismanagement. For example, an AI might label an important abnormality as “normal” (consistent with its low sensitivity, despite its high specificity) (1) potentially harming the patient if it goes undetected without a specialist's review. Although the authors do temper their claims by emphasizing that AI should complement rather than replace radiologists and note that human input is necessary for differential diagnoses (1), these caveats may be overshadowed by the study's headline conclusions.

Many commercial AI services promote a collaborative approach between their software and general practice veterinarians (15–19). However, general practitioners may lack the specialized radiology training needed to question or correct AI outputs. This raises the risk of overreliance—assuming high specificity implies comprehensive diagnostic accuracy—while missing subtle errors or overlooked findings (3). Consequently, the claim that “AI could reliably increase diagnostic availability but requires further human input” remains speculative by their data and may be undermined by the potential for systematic misinterpretation or clinical oversight.

Such speculation must be supported by rigorous, reproducible internal and external validation. Yet, critical details needed to replicate or verify this study are missing. The AI software is proprietary and “continuously updated and does not have version numbers” (1). Although the authors used the July 2022 version, the absence of fixed versioning or a detailed algorithm description prevents replication and raises concerns about whether future iterations will behave similarly. Transparency about training data is also limited, described broadly as a large, multi-institutional dataset (1). This lack of clarity conflicts with emerging expectations for medical AI. The CLAIM checklist, which the authors cite as followed, calls for detailed documentation of model architecture, dataset characteristics, and analytical methods (1, 20). However, shortcomings in versioning, uncertainty handling, and data/code sharing remain, with proprietary restrictions cited as justification (1). Compliance with such guidelines is not merely bureaucratic; it ensures that results are interpretable and reliable. The authors' inconsistent adherence to CLAIM guidelines and use of outdated references calls into question their familiarity with current veterinary radiology AI literature and relevant standards.

Such limitations stand in contrast to evolving international standards. In 2021, the FDA, Health Canada, and the UK MHRA jointly issued *Good Machine Learning Practice* (GMLP) principles, followed by guidance on *Transparency for Machine Learning-Enabled Medical Devices* (MLMDs), which emphasize performance monitoring, explainability, and version traceability as essential for safety and accountability (27, 28). Veterinary AI tools like SignalPet are not currently subject to these regulations, but such principles exist because trust in AI outputs requires ongoing monitoring for drift or changes over time. The authors also fail to clarify key analytical methods—such as how “median variance” was used to define case ambiguity—leaving readers with opaque results. In fields impacting patient care, such opacity, known as the “Black Box Problem,” is unacceptable (29). Reproducibility and transparent validation are not optional; they are fundamental to earning clinical trust.

The real-world context must be considered; AI performance in routine veterinary radiology practice will likely differ significantly from controlled study environments. Factors such as radiograph quality, patient positioning, uncommon conditions, and incidental findings could challenge an AI in ways not captured by a small retrospective sample. Where as SignalRAY^®^ has been marketed to general practice veterinarians who often experience more errors in radiographic techniques (21), the cases from this study are reported to come from the “institutional PACS;” appearing likely to be from either the Royal (Dick) School of Veterinary Studies or University of Veterinary Medicine Hannover, both teaching hospitals and likely practicing gold standard radiographic techniques.

The integration of AI into workflow also brings human-factor issues: How do veterinarians respond to AI suggestions? Do they over-rely on them or appropriately override them when needed? These questions have ethical weight. Over-reliance (automation bias) can occur if the AI is believed to be nearly as good as an expert—clinicians might defer to the AI even when it is wrong (6, 7). Under-reliance can also occur if the AI is not trusted at all. Calibrating this balance requires absolute clarity about the AI's limits. Overstating its performance tilts users toward excessive trust. As such, the study by Ndiaye et al. should be interpreted as a preliminary investigation rather than definitive proof of AI equivalence to radiologists, and readers should be concerned if this is the only published research assessing any form of performance validation. At this stage it is far safer to assume the AI is a fallible corroborator, rather than to assume it can match a specialist in diagnostic utility.

## Discussion

While the work of Ndiaye et al. is an important step in evaluating AI for veterinary imaging, its findings should be applied with great caution and concern. The study demonstrates the potential of current AI to assist in radiographic interpretation—possibly by confirming normal cases in a resource-limited setting—but it also highlights the limitations of both the technology and the evaluation methodology. Without a true gold standard and comparable pattern-recognition tasks between AI and radiologists, we cannot be sure that “AI accuracy” equates to clinical diagnostic accuracy (22). With a small, imbalanced sample, we cannot be sure the results would hold across the wide variety of patients and conditions encountered in practice (6). And without more detailed output from the AI, we know it cannot fulfill many tasks that human radiologists perform, from formulating differential diagnoses to making management recommendations. Crucially, the original study mischaracterizes AI's function as “interpretation” rather than pattern recognition, creating a critical mismatch with the radiologists' comprehensive interpretations. This fundamental mismatch in the tasks performed means that comparisons between the AI and radiologists are inherently flawed and arguably invalid.

For clinical veterinary researchers and practitioners, the takeaway is to remain critically aware of what AI publications are actually telling us. Exciting headlines about AI matching experts often gloss over the fine print. As this commentary has detailed, issues of bias, sample composition, and evaluation design can paint an overly rosy picture of an AI's capabilities. Ethically, we must avoid the trap of overstating AI performance—doing so risks patient care if veterinarians become overconfident with the use of an under-tested tool (6, 7). Given the identified limitations, this study should be considered preliminary at best, and should not serve as a basis for widespread adoption of this or similar AI tools in clinical practice. Instead, AI should be introduced into clinical practice gradually and under supervision, with continuous monitoring of its real-world performance and error modes. Far from discouraging the use of AI, a critical perspective ensures that when AI is used, it is done in a way that enhances diagnostic accuracy and efficiency rather than inadvertently undermining them. Ensuring rigorous standards in veterinary AI research is a shared responsibility that extends beyond investigators. Journals, editors, and peer reviewers must also enforce high levels of transparency, reproducibility, and methodological integrity before publication (23, 24). Peer reviewers play a vital role in this process, critically assessing claims and requiring detailed, well-justified technical explanations (24, 25). In parallel, professional veterinary associations have called for robust ethical oversight to protect scientific integrity and animal welfare (26).

In the veterinary radiology community—as in human radiology—there is genuine enthusiasm for the benefits AI might bring, such as faster turnaround, enhanced screening, and decision support. To realize these benefits, we must demand robust evidence of safety and efficacy. This study falls short of these essential standards, and caution should prevail in interpreting and implementing its findings. Until then, AI remains a promising adjunct, not a replacement, for veterinary radiologists.

To move the field forward, veterinary research must address current gaps through larger, multi-center studies using validated ground truths, like consensus readings supported by clinical outcomes. Rigorous statistical analysis, adherence to reporting standards (e.g., CLAIM), and robust peer review are essential to ensure transparency and reproducibility. AI research must be held to the same high standards as any clinical investigation to generate reliable evidence for clinical decision-making. Transparent collaboration, including both internal and external validation of algorithms, is critical—along with disclosure of training data sources. A commitment to this level of rigor, informed by critical appraisal of studies like Ndiaye et al.'s, will help ensure that veterinary AI advances responsibly, with animal welfare at the forefront.
